# Interleukin-18 in metabolism: From mice physiology to human diseases

**DOI:** 10.3389/fendo.2022.971745

**Published:** 2022-10-12

**Authors:** Emmanuel Somm, François R. Jornayvaz

**Affiliations:** ^1^ Service of Endocrinology, Diabetes, Nutrition and Therapeutic Patient Education, Department of Internal Medicine, Geneva University Hospitals, Geneva, Switzerland; ^2^ Department of Cell Physiology and Metabolism, University of Geneva, Geneva, Switzerland; ^3^ Diabetes Center, Faculty of Medicine, University of Geneva, Geneva, Switzerland

**Keywords:** obesity, diabetes mellitus, NAFLD, NASH, gut microbiota, inflammation, interleukin-18

## Abstract

Interleukin-18 (IL-18) is a classical member of the IL-1 superfamily of cytokines. As IL-1β, IL-18 precursor is processed by inflammasome/caspase-1 into a mature and biologically active form. IL-18 binds to its specific receptor composed of two chains (IL-18Rα and IL-18Rβ) to trigger a similar intracellular signaling pathway as IL-1, ultimately leading to activation of NF-κB and inflammatory processes. Independently of this IL-1-like signaling, IL-18 also specifically induces IFN-γ production, driving the Th1 immune response. In circulation, IL-18 binds to the IL-18 binding protein (IL-18BP) with high affinity, letting only a small fraction of free IL-18 able to trigger receptor-mediated signaling. In contrast to other IL-1 family members, IL-18 is produced constitutively by different cell types, suggesting implications in normal physiology. If the roles of IL-18 in inflammatory processes and infectious diseases are well described, recent experimental studies in mice have highlighted the action of IL-18 signaling in the control of energy homeostasis, pancreatic islet immunity and liver integrity during nutritional stress. At the same time, clinical observations implicate IL-18 in various metabolic diseases including obesity, type 1 and 2 diabetes and nonalcoholic fatty liver disease (NAFLD)/nonalcoholic steatohepatitis (NASH). In the present review, we summarize and discuss both the physiological actions of IL-18 in metabolism and its potential roles in pathophysiological mechanisms leading to the most common human metabolic disorders, such as obesity, diabetes and NAFLD/NASH.

## Introduction

Interleukin-18 (IL-18) is a member of the IL-1 superfamily of cytokines structurally similar to IL-1beta (IL-1β) (1, 2). Originally, Kupffer cell (liver-resident macrophage) was described as the main source of IL-18 (3). Nevertheless, many other cell types, including both hematopoietic cells and non-hematopoietic cells (such as intestinal epithelial cells, keratinocytes, endothelial cells) have also the potential to produce IL-18 in the basal state or under stimulation (3). IL-18 is regulated at the transcriptional, post-transcriptional and post-translational levels. The *IL18* gene, which contains 7 exons, is located on chromosome 11 in humans and chromosome 9 in mice (4). The human *IL18* promoter presents several single nucleotide polymorphisms (SNPs) at the 5′-end impacting gene transcription (5). *IL18* gene expression could also be regulated by miRNAs (6). IL-18 is characterized by a specific mechanism of cellular production (partly shared with IL-1β). In contrast to cytokines expressed and subsequently secreted thank to their signal peptide, IL-18 is stored in the cytosol of its producing cells as a biologically inactive precursor (pro-IL-18) (4, 7). Pro-IL-18 requires post-translational processing to become biologically active and released extracellularly, meaning that constitutive or stimulated expression of IL-18 does not necessarily imply its secretion. Caspase-1 (initially named IL-1β-converting enzyme) is the major IL-18-processing enzyme, acting in large cytoplasmic multiprotein complexes named inflammasomes (8, 9). Inflammasomes are activated by specific stimuli and are composed of a sensor molecule, an adaptor protein, and an effector molecule responsible for precursor cleavage. Of note, caspase-1 requires autolysis to be cleaved into active caspase-1 which converts pro-IL-18 into mature IL-18. In addition, active caspase-1 cleaves gasdermin D and liberates a pore-forming domain allowing the release of mature IL-18 (10). The nucleotide-binding oligomerization domain leucine rich repeat and pyrin domain containing 3 (NLRP3) inflammasome was originally demonstrated to be a cytoplasmic platform necessary for caspase-1 activation (11). In some cells (such as blood-derived macrophages), NLRP3 inflammasome activation requires two signals: priming (generally mediated by Toll-like receptor [TLR]) and activation (triggered by various cellular responses including endogenous or exogenous particulates/microcrystals/metabolites/mitochondrial oxidized DNA). Bacterial or viral components are also sensed by inflammasome subunits, promoting inflammasome assembly and caspase-1-mediated IL-18 secretion (12). In Kupffer cells, LPS can trigger IL-18 secretion without priming (13). IL-18 maturation can also occur through the activation of other inflammasomes, such as NLRP6 or NLRP9. Other proteases, including caspase-8 (an apoptosis-initiating protease), proteinase 3, chymase or granzyme B are also involved in conversion of pro-IL-18 into mature IL-18 independently of NLRC4, NLRP3, or caspase-1 (3, 14). IL-18 signals through the IL-18 receptor (IL-18R), belonging to the IL-1R family. IL-18R is composed of the IL-18Rα chain (IL-18R1/IL-1Rrp) and the IL-18Rβ chain (IL-18R accessory protein/IL-1RAcPL) (4). Binding of IL-18 to IL-18Rα and IL-18Rβ chains forms a trimer. The intracellular region of IL-18R contains a TIR domain (analog to TLR) that binds MyD88 and initiates a signal transmission into the cell (15). MyD88 recruits IRAK1 and IRAK4 (16, 17), followed by binding to TRAF6, degradation of inhibitor of κB (IκB), phosphorylation and nuclear translocation of p65/p50 NF-κB (18). Other kinases, including the Mitogen-Activated Protein Kinase (MAPK) cascade of Extracellular Signal-regulated Kinase (ERK), c-jun N-terminal kinase (JNK), and p38 are also activated and implicated in IL-18 signaling (19). Together, these signals induce IFN-γ production or cell proliferation. IL-18 also induces the phosphorylation and activation of phosphatidylinositol-3 kinase (PI3K)/Akt/S6 and mammalian target of rapamycin (mTOR) (20). This signaling enhances the proliferation and survival of Natural Killer (NK) cells. IL-18R expression is stimulated by IL-12, IFN-α or Signal Transducer and Activator of Transcription (STAT)4 in T cells and NK cells (3). IL-18R is also expressed in non-immune cells (epithelial cells, nerve cells, etc…) in which IL-18 signaling is involved in cell survival and differentiation. Surprisingly, IL-18Rα also binds to IL-37 (IL-1F7), preventing IL-18 signaling. IL-18Rα/IL-37 complex binds to IL-1R8 (TIR8/SIGIRR), promoting an anti-inflammatory effect through STAT3 (21). In circulation, IL-18 binds to the IL-18 binding protein (IL-18BP) with high affinity (higher affinity than IL-18R), leaving only a small fraction of free IL-18 able to trigger receptor-mediated signaling. In consequence, IL-18BP can be considered as a negative regulator of the IL-18 signaling pathway (22, 23). Immunologic functions of IL-18 are pleiotropic and include stimulation of IFN-γ production, activation of basophils and mast cells, allergic inflammation, defense against extracellular pathogens, helminth infection and intracellular pathogens (bacteria, protozoan, virus) (3).

If the role of IL-18 in inflammatory and infectious diseases is well established, recent experimental studies in mice have involved IL-18 signaling in the control of energy homeostasis, pancreatic islet immunity and liver integrity during nutritional stress. At the same time, clinical observations have implicated IL-18 in various metabolic diseases including obesity, type 1 (T1D) and type 2 (T2D) diabetes, and nonalcoholic fatty liver disease (NAFLD)/nonalcoholic steatohepatitis (NASH). In the present review, we summarize and discuss the physiological roles of IL-18 in metabolism and its potential involvement in pathophysiological mechanisms leading to the most common human metabolic disorders, such as obesity, diabetes, and NAFLD/NASH.

## Role of IL-18 in obesity

Due to behavioral, environmental, and sometimes genetic factors, the prevalence of obesity has risen to unacceptable levels worldwide (24). Obesity is associated with a shortened life span, predisposing to T2D, cardiovascular diseases, liver disorders, and cancers among others (24). In this pathophysiological context, a huge research effort has been brought to the study of cytokines (including IL-18) produced by the adipose tissue and upregulated during obesity. Beyond inflammation, these ‘‘adipokines’’ are also involved in the crosstalk between metabolic organs, acting on several physiological processes (such as energy homeostasis or glucose/fat metabolism) involved in obesity onset (25).

### Central action of IL-18 in obesity and behavior

First evidence for a role of IL-18 in the regulation of body weight and fat mass comes from the phenotyping of mice globally deficient in IL-18. IL-18^-/-^ mice displayed primary hyperphagia leading to obesity and insulin resistance in the liver, adipose tissue, and muscle (26). Similar hyperphagia was observed in mice deficient in IL-18R and in mice overexpressing IL-18BP, while central administration of recombinant IL-18 (rIL-18) inhibited food intake and reversed hyperglycemia in IL-18^-/-^ mice (26). Both central and peripheral administration of IL-18 suppressed appetite and weight regain in food-deprived mice (27). In animal models of binge eating, a down-regulation of the IL-18/IL-18R system (but increased expression of IL-18BP) was specifically observed in the preoptic and anterior-tuberal region of the hypothalamus (28). In contrast, food restricted animals exhibited increased IL-18 expression (28). In humans, plasma IL-18 levels were significantly decreased in patients with anorexia nervosa and circulating IL-18 levels correlated to body mass index (BMI) in controls, but not in anorexic patients (29). Additional studies have attempted to elucidate the mechanisms of IL-18 action on food intake in the central nervous system (CNS).

Firstly, investigations concerning the distribution of IL-18Rα in the mouse brain allowed exploring the exact sites of central IL-18 action. *In situ* hybridization combined with immunohistochemistry revealed that IL-18Rα is expressed in neuronal cell bodies, as well as on their dendrites, throughout the brain, with particularly high levels in regions involved in metabolic control such as the hypothalamus and the thalamus but also in other regions of the limbic system such as the hippocampus, the amygdala, and the cerebral cortex (30).

Secondly, cellular mechanisms underlying the anorexigenic effects of IL-18 have also been deeply investigated. A high expression of both subunits of the IL-18R was detected in the bed nucleus of the stria terminalis (BST), a region of extended amygdala known to influence feeding *via* its projections to the lateral hypothalamus (LH) (31). Local injection of rIL-18 in this area significantly reduced c-fos activation and food intake (31). In BST brain slices, IL-18 reduced the excitatory input on neurons through a presynaptic mechanism (31). This effect was cell-specific, only observed in Type III GABAergic neurons located in the juxtacapsular BST. In consequence, IL-18 increases the firing of glutamatergic LH neurons through a mechanism of disinhibition (31).

In addition, to hyperphagia, adult IL-18^-/-^ mice gained 2- to 3-fold more weight than wild-type mice per unit of food consumed (low- or high-fat diet) (27). This suggests that in addition to its anorexic action, IL-18 limits food efficiency. Accordingly, indirect calorimetry revealed a reduced energy expenditure in IL-18^-/-^ mice, in association with an increased respiratory exchange ratio (RER), suggesting a preferential oxidation of carbohydrate at the expense of fat (27). The reduction in energy expenditure of IL-18^-/-^ mice was seen across fasting/feeding conditions, low/high-fat diets, low/high levels of physical activity and times of day, suggesting an underlying action of IL-18 on basal metabolic rate (32). The circadian amplitude of energy expenditure, but not those of RER, food intake, or motor activity, was also blunted in IL-18^-/-^mice (32). In accordance, hepatic gene expression of circadian regulators [such as circadian locomotor output cycles kaput (CLOCK), brain and muscle Arnt-like protein (BMAL1), and period circadian clock 2 (PER2)] was also altered in IL-18^-/-^ mice (33).

**Figure 1:**
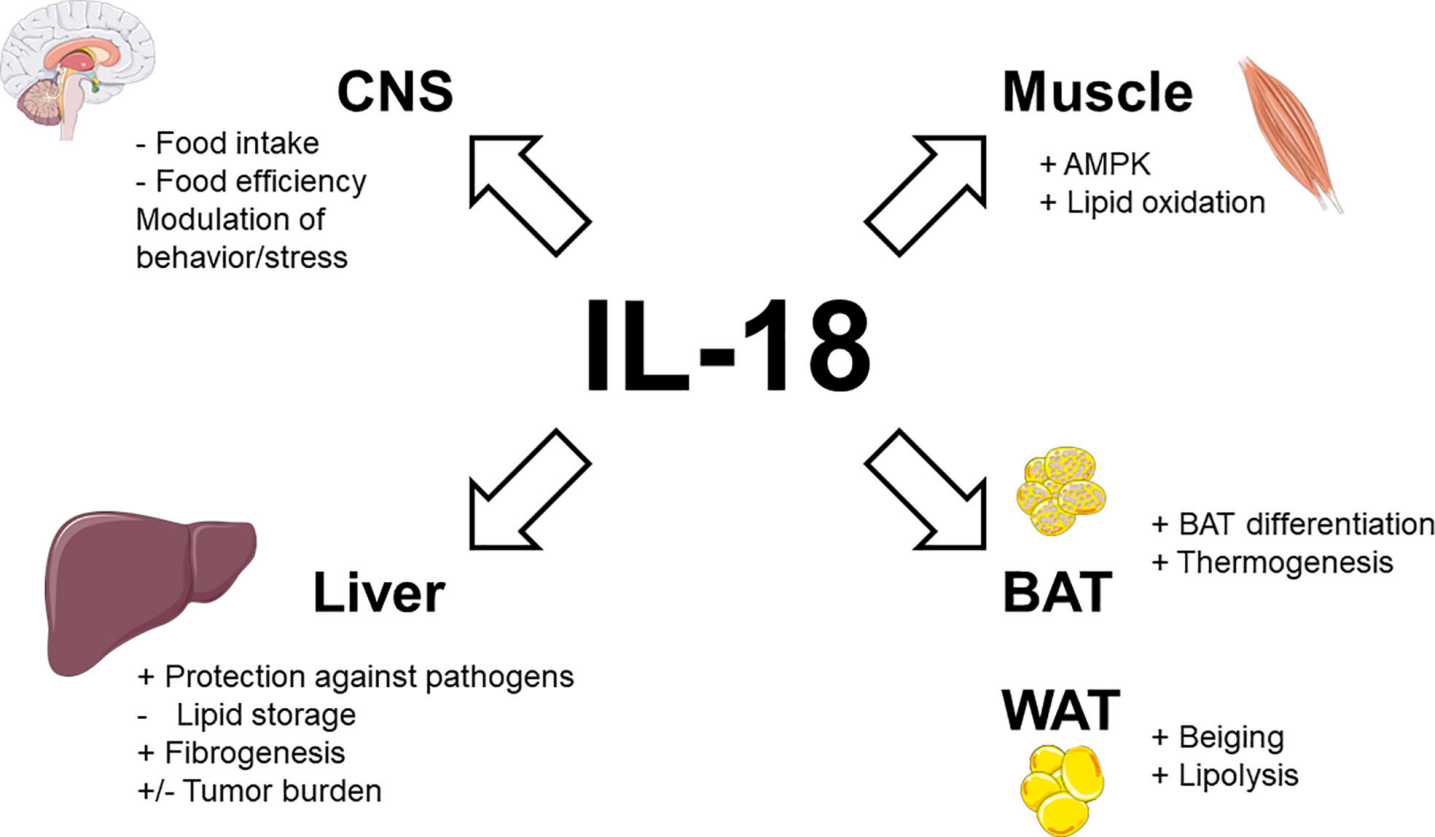
Physiological actions of IL-18 signaling in metabolic tissues. Stimulatory effects are indicated by plus signs (+), inhibitory effects by minus signs (−). Figure produced using illustrations from Servier Medical Art (smart.servier.com) under Creative Commons Attribution 3.0 unported license.

Taken together, these data demonstrate that endogenous IL-18 not only suppresses appetite but also promotes energy expenditure and lipid oxidation (as illustrated in **Figure 1**).

Outside the metabolic field, IL-18^-/-^ mice also exhibited a reduction in depressive-like behavior and a decreased expression of neuroendocrine genes in the amygdala (34). In humans, association between polymorphisms in the IL-18 gene (resulting in higher IL-18 production) and depressive behavior have been reported (35, 36). In addition, other observations also involved IL-18 in stress responses and the hypothalamic-pituitary axis (37, 38–43).

### Peripheral action of IL-18 in obesity

In addition to its role in the inhibition of food intake, some evidence has implicated peripherally-produced and -acting IL-18 in the regulation of body weight homeostasis and fat mass accretion.

#### IL-18 in brown adipose tissue

*In vitro*, brown adipocyte precursors isolated from IL-18^-/-^ mice showed an impaired differentiation featured by increased lipid accumulation and decreased gene expression of type 2 iodothyronine deiodinase when compared to those of control mice (44). Similar findings were observed *in vivo*, since the BAT of IL-18^-/-^ mice spontaneously accumulated fat and overexpressed Apoc3 and Insig1 (44). Treatment with rIL-18 reduced the size and number of fat droplets in BAT of IL-18^-/-^ mice (44). The surrounding perivascular adipose tissue (PVAT) of IL-18^-/-^ mice exhibited a conversion from brown adipose tissue-like features to white adipose tissue-like features, impacting the aorta physiology (45). In line with these observations, IL-18^-/-^ mice failed to develop diet-induced thermogenesis as shown by non-induction of uncoupling protein-1 (UCP-1) in BAT and inguinal white adipose tissue (iWAT) (46). This defect could result from non-activation of some IL-18R expressing subpopulations of group 2 innate lymphoid cells (ILC2s) (47), an immune cell type known to promote adipocyte beiging (48). Surprisingly, IL-18R^-/-^ mice were overweighted on standard chow diet but appeared to resist to high-fat diet (HFD)-induced obesity or cold exposure-induced hypothermia (46), suggesting the absence of BAT dysfunction. This discrepancy between IL-18^-/-^ and IL-18R^-/-^ mice suggests that a more complex signaling system than IL-18/IL-18R interaction could be involved in the adipose thermogenic action of IL-18 (46). In this way, the anti-inflammatory IL-37 which binds IL-18Rα/IL-1R8 heterodimer has not yet been identified in mice (49).

#### IL-18 in white adipose tissue

IL-18 is produced in WAT. In mice and humans, obesity was associated with increased IL-18 levels in WAT, which contribute to systemic concentrations (50–55). Expression of the IL-18R and the IL-18BP was also observed in human WAT, mirroring that of IL-18 (56). IL-18 gene expression was evident in human subcutaneous and visceral adipose tissues (56), but a higher secretion levels of IL-18 was observed in explant from visceral WAT, possibly due to increased inflammasome and caspase-1 activation in this intraperitoneal depot (57). The cellular source of IL-18 in WAT was a matter of debate since several studies reported that expression of IL-18 occurs in both mature adipocytes and the stromal vascular fraction (56), while other stated that most of the IL-18 released by explants of human adipose tissue are derived from the non-fat cells, not from the adipocytes (58). In this way, IL-18 expression is similar in mesothelial cells and the stromal vascular fraction (59). Other observations showed that IL-18 was immunolocalized in WAT neutrophils and mast cells, but not in macrophages or in adipocytes (59). In humans, IL-18 expression was increased in adipose tissue from HIV-associated lipodystrophic patients (60) and a haplotype associated with lower IL-18 levels was associated with a higher body mass index (61). Weight loss after bariatric surgery (62–64), or after lifestyle changes (53, 65), resulted in decreased IL-18 concentrations in the blood.

Paracrine effects of IL-18 in WAT could contribute to regulation of adiposity. Through an activating mutation in the inflammasome NLRP1, it has been shown that IL-18 triggers lipolysis in WAT (66). Interestingly, this action could explain susceptibility to obesity/diabetes of C57BL/6 mice compared to the healthy metabolic status of Balb/c mice. In fact, while C57BL/6 mice harbor the NLRP1b2 allelic inflammasome variant, Balb/c mice harbor the NLRP1b1 inflammasome which produces more important IL-18 levels, resulting in higher lipolysis, reduced WAT inflammation and improved insulin sensitivity (67). Mechanistically, IL-18 promoted adipose hormone-sensitive lipase (HSL) phosphorylation and activation (67). IL-18 also enhanced insulin-mediated glucose uptake in adipocytes and could counteract the suppression of glucose uptake caused by Tumor Necrosis Factor (TNF)-α in 3T3-L1 adipocytes (68). Underlying mechanisms involved phosphorylation of protein kinase B (Akt) and downregulation of phosphorylated p38 MAPK (68).

Obesity-related upregulation of IL-18, which experimentally limits food intake and food efficiency while promoting energy expenditure and fat oxidation could appear surprising. However, as for leptin, obesity could reflect a state of IL-18 resistance. In this way, it is interesting to note that phosphorylation and then activation of STAT3 is triggered both following IL-18 signaling (27) and leptin signaling (69, 70), suggesting a crosstalk/convergence of these signaling pathways. In accordance with this perspective, monocytes from obese patients (leptin resistant) were also desensitized to IL-18 (71). The stimulatory activity of IL-18 signaling on brown adipocyte differentiation and thermogenesis, as well as on white adipocyte transdifferentiation (into brown-like adipocyte-beiging) and lipolysis are summarized in **Figure 1**.

#### IL-18 in skeletal muscle

Immunohistochemistry demonstrated that IL-18 is solely expressed by type II (fast-twitch) fibers in different human skeletal muscles (72). This suggests that the level of daily muscle activity does not influence basal IL-18 expression, which is rather implicated in normal physiology (72). A basic study in mice deficient in IL-18 signaling confirmed that the action of IL-18 in skeletal muscle could contribute to its anti-obesity effect. In addition to increased weight gain, IL-18R-/- mice display ectopic lipid deposition, inflammation, and reduced AMP-activated protein kinase (AMPK) signaling in skeletal muscle (73). Conversely, electroporation of IL-18 into normal skeletal muscle activated AMPK and concomitantly inhibited HFD-induced weight gain (73). *In vitro*, treatment of myotubes and skeletal muscle strips with IL-18 also activated AMPK and increased fat oxidation (73). Patients with human immunodeficiency virus (HIV)-lipodystrophy had lower levels of muscular IL-18 and IL-18R gene expression (74). These low levels of IL-18 correlated to high muscular levels of deleterious lipid species as ceramides and sphingosine-1P and increased levels of triglycerides in circulation (74). The combined activations of AMPK and lipid oxidation by IL-18 signaling in skeletal muscle are shown in **Figure 1**.

## Role of IL-18 in diabetes

Type 1 diabetes mellitus (T1D) is an endocrine disorder in which pancreatic β-cells stop producing insulin, typically due to autoimmune destruction (75). Incidence peaks in puberty/early adulthood, but onset can occur at any age (75). Type 2 diabetes mellitus (T2D) is one of the most common illnesses encountered by internists (76). Featured by insulin resistance, T2D is increasing worldwide due to populations aging and obesity pandemic (76). Although diabetes care is improving by many measures, complications are still common, including visual loss, amputation, atherosclerotic disorders or end-stage renal disease (76). Management of both T1D and T2D should focus on optimizing glucose control to reduce acute consequences (such as diabetic ketoacidosis or hyperosmolarity) and long-term complications (including microvascular and macrovascular diseases) (75). Experimental and clinical evidence has involved IL-18 in the onset/progression of both T1D and T2D.

### IL-18 in T1D

Innate immunity contributes to the induction and amplification of the immune response inducing β-cells loss in T1D (77). In addition, a crosstalk between immune cells and stressed β-cells is mediated by cytokines and others immunogenic signals delivered by stressed β-cells (77). IL-18 has been detected in rodent pancreatic β-cells (78, 79), exhibiting only a minor stimulatory effect on insulin secretion (80). If a protective role has been early conferred to IL-18 in diabetes onset in Non Obese Diabetic (NOD) mice [in link with impaired progression from Th2- to Th1-dependent insulitis (81)], further works have globally attributed a deleterious role of IL-18 in T1D. IL-18 was detected in NOD mouse pancreatic islets during early stages of insulitis (82), mediating islet injury (83). In response to the alkylating agent cyclophosphamide, macrophages from NOD mice presented an increase in IL-18 gene expression closely associated with diabetes development, while macrophages from Balb/c mice did not (82, 84). IL-18 has been implicated in the expansion of islet-destructive T-cells during pre-diabetes. In fact, IL-18 expanded pathogenic T-cells in the periphery of NOD mice (85), while IL-18^-/-^ mice exhibited a reduced T-cell turnover, an increased prevalence of naïve/quiescent T-cells and less effector T-cells, resulting in disease protection (85). In addition, islet-reactive T-cells failed to become activated and expanded in the lymphoid organs of IL-18^-/-^ mice (85). Systemic administration of IL-18 also promoted diabetes development in young NOD mice (86), while endogenous IL-18 was required to observe the full diabetogenic effect of streptozotocin in C57BL/6 mice (87, 88).

Clinical evidence also confirmed a role for IL-18 in T1D. From a genetic perspective, the genomic loci idd2, associated with T1D, maps in close proximity to the IL-18 gene (82). Positive association between T1D and polymorphisms in the promoter of IL-18 gene (leading to increased IL-18 gene expression) were found in some studies (89, 90), but not in others (91–93). IL-18 serum levels and IL-18 protein expression in pancreatic islets were increased selectively in T1D patients (94–96). IL-18 was also detected in islets infected with enterovirus in pancreas of patients presenting a fulminant T1D (97).

### IL-18 in T2D

Several properties of IL-18 previously described, including stimulation of insulin-mediated glucose uptake (68), activation of AMPK (73) or phosphorylation of STAT3 suggest a beneficial role for IL-18 in glucose homeostasis. Nevertheless, numerous clinical studies have described an upregulation of IL-18 circulating levels in T2D. Notably, patients with prediabetes have higher levels of IL-18 compared to obese normoglycemic controls (98). Circulating IL-18 was increased in patients with T2D (99–101), independently of a generalized pro-inflammatory state (102). This association was independent of usual risk factors, including BMI and other adipokines levels (103, 104). In addition, circulating IL-18 levels were positively correlated with the Homeostasis Model Assessment of Insulin Resistance (HOMA-IR) index (105) and glucose intolerance, independently of BMI or age (104, 106, 107). Conversely, a decrease in IL-18 was an independent factor for the improvement of β-cell function in T2D (108). A polymorphism of the IL-18 gene associated with increased circulating levels of IL-18 has been linked to impaired insulin sensitivity (109). Similarly to insulin, high blood IL-18 levels observed in T2D could reflect a state of IL-18 resistance.

## Role of IL-18 in NAFLD/NASH

Obesity and T2D are frequent causes of NAFLD, the most common hepatic disease in industrialized countries (110, 111). A significant proportion of patients with NAFLD develop a state of hepatic inflammation (NASH), which can lead to fibrosis and cirrhosis, potentially resulting in hepatocellular carcinoma (HCC) (112). Whilst changes in eating habits, weight loss or physical activity have beneficial effects on liver steatosis, no efficient pharmacologic treatment are available to limit the progression of NAFLD, NASH and fibrosis (113). Some clinical observations as well as basic studies in rodents suggested a role for IL-18 in the onset/progression of NAFLD/NASH. The next sections will summarize how IL-18 can directly or indirectly act on the liver status before reviewing its implication in hepatic carcinogenesis.

### Direct effect of IL-18 on the liver

IL-18 is mostly produced by macrophages in the liver (114, 115), mediating hepatic defense against bacteria, parasites, virus, and drug-induced injuries (116–119). Ability of IL-18 to activate NK cells, inducing apoptosis of infected/damaged hepatocytes through the Fas ligand (FasL) pathway (115, 120), is central in this context. Nevertheless, IL-18 signaling required a fine-tuning. Uncontrolled IL-18 signaling due to loss-of-function in IL-18BP could result in massive death of human hepatocytes *in vitro* and in patients (115). Metabolically, toxic lipids activate the NLRP3 inflammasome and IL-18 production in different NAFLD/NASH murine models (121–123). Several experimental evidence suggest that IL-18 is involved in both steatotic and fibrotic processes.

Observations in rodents suggest that IL-18 could suppress hepatic lipid deposition. NLRP1^-/-^ mice (with low IL-18 levels) spontaneously developed hepatic steatosis, a situation aggravated on HFD (66). In contrast, mice with constitutively activated NLRP1 (with high IL-18 levels) were devoid of lipid vacuoles in the liver, and depletion in IL-18 reversed this protective effect (66, 124). Obese C57BL/6 mice (naturally harboring the NLRP1b2 allele-resulting in low IL-18 levels) have increased hepatic steatosis when compared to obese NLRP1b1 transgenic C57BL/6 mice or Balb/c mice (naturally harboring the NLRP1b1 allele-resulting in high IL-18 levels) (67). High levels of IL-18 also mediated the reduction in hepatic steatosis observed in mice with a conditional deficiency in Src homology-2 domain-containing protein tyrosine phosphatase-2 (SHP2) in macrophages (125). In accordance, administration of exogenous IL-18 counteracted steatohepatitis in mice on HFD (66) while IL-18^-/-^ mice exhibited hepatic steatosis, insulin resistance, increased expression of gluconeogenic genes and defective phosphorylation of STAT3 (26). Several explanations have been argued to explain this NAFLD/NASH phenotype of IL-18^-/-^ mice. The initial study suggested that primary hyperphagia and resulting obesity were the cause of steatosis in IL-18^-/-^ mice (26). Later work showed that IL-18^-/-^ mice developed hypercholesterolemia and hypertriglyceridemia before the manifestation of obesity (33) suggesting a primary effect of IL-18 on the liver. In accordance, hepatic transcriptional changes were observed in the liver of IL-18^-/-^ mice before obesity onset (33). Finally, a role for altered gut microbiota composition has been advanced to explain steatosis in IL-18^-/-^ mice (126). Hepatic insulin-resistance resulting from IL-18 deficiency seems to be sex-hormone-dependent (127). In addition, IL-18R^-/-^ mice (but not IL-1R^-/-^ mice) were protected from precocious dietary liver damage, possibly due to silencing of early pro-inflammatory genes initiating NASH (128).

Other basic studies suggest that IL-18 could enhance hepatic fibrosis. In fact, IL-18 induce multiple functional changes in Hepatic Stellate Cells (HSCs), the resident perisinusoidal cells orchestrating the deposition of extracellular matrix (ECM) in normal and fibrotic liver (129). *In vitro*, monosodium urate-induced inflammasome activation led to overexpression of TGF-β and collagen 1 in primary mouse HSC and HSC line (130). *In vivo*, NLRP3^-/-^ mice had reduced chemically-induced liver fibrosis (130) while conditional NLRP3 knock-in mice expressing an hyperactive NLRP3 present HSC activation with increased collagen deposition in the liver (131). These changes were only partially attenuated by treatment with an interleukin-1 receptor antagonist suggesting that beyond IL-1β, IL-18 is also involved in fibrogenesis (131). In this way, adoptive transfer of CD4^+^ T cells from IL-18 transgenic mice (but not from wild-type littermates) in SCID mice resulted in massive periportal fibrosis (132). The direct consequences of IL-18 signaling on liver physiology, including protection against pathogens, inhibition of lipid storage and stimulation of fibrogenesis are detailed in **Figure 1**.

Clinically, circulating IL-18 levels were associated with increased liver injury markers and portal fibrosis in obese subjects with NAFLD (133), as well as with plasma concentrations of liver injury markers in healthy subjects (134). Patients with NAFLD had significantly higher IL-18 and IL-18/IL-18BP ratio compared with healthy controls (135). Plasma levels of IL-18 and IL-18BP were elevated in chronic liver diseases such as cirrhosis, correlating with inflammation, liver injury and severity of the disease (136). However, IL-18BP levels may not be sufficient to counteract the overwhelming pro-inflammatory response in end stage liver disease (136). Genetically, IL-18 variants, resulting in higher IL-18 levels, were significantly associated with chronic liver diseases (including cirrhosis) in the overall population (137).

Taken together, these results suggest that a physiological/limited amount of IL-18 exhibits interesting anti-steatotic properties while IL-18 excess could be deleterious for liver integrity.

### Indirect effect of IL-18 on the liver through the gut-liver axis

The intestinal mucosal and portal vein enables transport of gut-derived products directly to the liver and in turn, the liver secretes bile and other compounds in the intestine. This direct interaction, concomitantly allowing nutrients to directly reach the liver and limiting the dissemination of microbes and toxins to the systemic circulation is called the ‘‘gut-liver axis’’ (138). Dysbiosis and gut leakiness play a critical role in the development of NAFLD/NASH (139, 140). IL-18 controls the gut-liver axis at multiple interconnected levels, including the maintenance of intestinal epithelial barrier, the production of intestinal mucus and the production of intestinal Anti-Microbial Peptide (AMP), all impacting gut microbiota composition.

#### IL-18 in maintenance of intestinal epithelial barrier

Several evidence confer a deleterious role to IL-18 in gut epithelium integrity. Systemic administration of IL-12 and IL-18 to wild-type mice induced intestinal mucosal inflammation (141) while administration of IL-18BP reduced intestinal inflammation and ulceration (142). In addition, IL-18 overproduction in the mucosa exacerbated infiltration of macrophages and colitis (143) in mouse models of gut inflammation. In line, mice with a genetic deletion of IL-18 or its receptor IL-18R1 in intestinal epithelial cells were protected from chemically-induced mucosal damage (144). In humans, IL-18 is produced by gut epithelial cells and macrophages and this production was increased during inflammatory bowel diseases (145–148). Nevertheless, other studies have suggested a protective role for physiologic amount of IL-18 on intestinal epithelium, in particular through its crosstalk with IL-22. In fact, IL-18 could increase the ratio of IL-22/IL-22BP, which exerts protective properties during the peak of gut epithelial damage (149). In turn, a study in ileum organoids showed that IL-22 transcriptionally activates epithelial IL-18 (150). In colitis mouse models, inflammasome activation led to an increase in both IL-18 production and mucosal barrier integrity, resulting in a decreased hepatic bacterial load (151). Epithelium-derived IL-18 seems to contribute to epithelial proliferation through induction of stem cell genes (150). In this way, it has been suggested that IL-18 polymorphisms known to reduce IL-18 mRNA and protein levels may be involved in the susceptibility to Crohn’s disease (152).

#### IL-18 in the production of intestinal mucus

Excessive IL-18 signaling through genetic deletion of IL-18BP resulted in loss of mature mucus-producing goblet cells associated with colitis (144). Goblet cells defect observed in IL-18BP^-/-^ mice was rescued by concomitant deficiency for IL-18R1 in intestinal epithelial cells, demonstrating the autocrine/paracrine deleterious action of uncontrolled IL-18 production (144). Mechanistically, it seems that IL-18 excess inhibited the transcriptional program of goblet cells development (144). However, it has been recently observed that IL-18 could stimulate mucin secretion from goblet cells during Escherichia coli infection (150).

#### IL-18 in the production of intestinal AMP

RNA-sequencing of colon from IL-18^-/-^ mice, as well as administration of IL-18 to germ-free colon explants or mice, revealed that non-hematopoietic IL-18 induced AMP, in particular intelectin 1 (ITLN1), resistin-like molecule β/FIZZ2 (RELMβ) and angiogenin-4 (ANG4) in a NF-κB-dependent manner (151). This was confirmed by another study showing that IL-18 induces both Paneth cell-related AMP (ITLN1, ANG4) and Paneth cell-specific AMP (lysozyme, cryptdin) in a STAT-dependent manner (150). In contrast, IL-18^-/-^ mice exhibited reduced mRNA levels of AMP and lysozyme-containing Paneth cells (150). Defect in IL-18 and AMPs due to intestinal NLRP6 inflammasome-deficiency resulted in a specific gut microbiota (151). Similarly, mice deficient in the AIM2 inflammasome had few colonic levels of IL-18, low expression of AMP, and were highly susceptible to colitis and microbiota dysbiosis (in particular Escherichia coli enrichment) (153). Recently, confocal microscopy studies revealed that intestinal neurons produce IL-18 (154). Deletion of IL-18 from the enteric neurons, but not from immune or epithelial cells, made mice susceptible to invasive Salmonella infection (154). Mechanistic approaches showed that enteric neuronal IL-18 was specifically required for homeostatic goblet cells AMP production (154). If IL-18 can modulate AMPs and impact gut microbiota, the reciprocal action is also true. In fact, both germ free mice and wild-type mice transplanted with IL-18^-/-^ mice microbiota exhibited a suppression of colonic IL-18 levels (151). Metabolomic screening revealed that microbiota-derived taurine enhanced (while histamine and spermine suppressed) NLRP6 inflammasome-induced IL-18 secretion (151).

#### IL-18 in the regulation of gut microbiota composition

Several studies have shown that IL-18 modulates gut microbiota composition. NLRP1 and downstream IL-18 reduced the amount of beneficial butyrate-producing Clostridiales in the gut, aggravating experimentally-induced colitis (155). In NLRP3^-/-^ mice, potentially pathogenic members of Enterobacteriaceae (including Citrobacter, Proteus or Shigella) were over-represented in the gut microbiota (156). Microbiota from NLRP6^-/-^ and IL-18^-/-^ mice on methionine-choline deficient (MCD) diet was characterized by increased proportion of Prevotellaceae and TM7 phylum (126). A significantly increased levels of Akkermansia muciniphila (a bacterial strain with ability to degrade mucus) has also been observed in IL-18^-/-^ mice (157). This central role of IL-18 in microbiota maintenance impacts, in consequence, liver metabolism. In fact, increased proportion of Prevotellaceae and TM7 phylum in the microbiota from IL-18^-/-^ mice led to an exacerbated influx of TLR4 and TLR9 agonists into the portal circulation, leading to hepatic overexpression of TNF-α that drives NASH progression (126).

**Figure 2:**
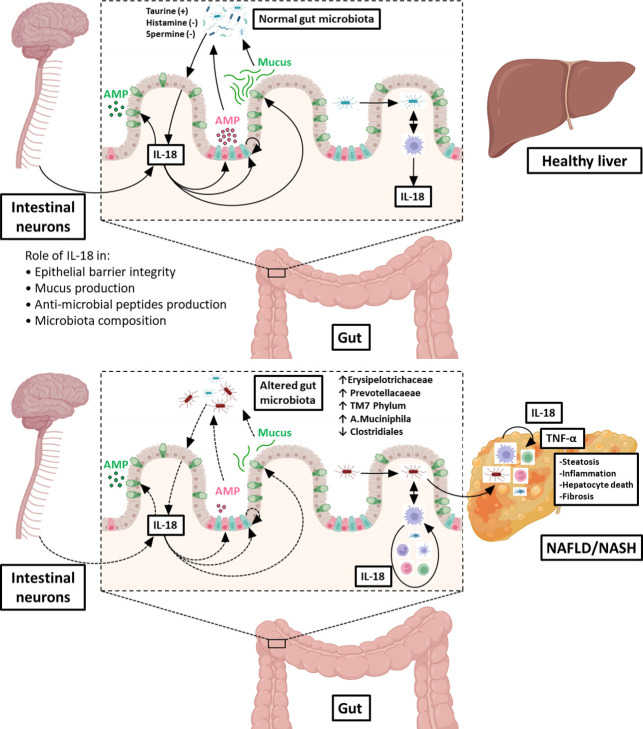
Role of IL‐18 in the gut microbiota balance and consequences on the gut-liver axis. Under physiological conditions (upper illustration), IL‐18 is produced by intestinal epithelial cells (in light pink) and is regulated by microbiota-derived metabolites (such as taurine, histamine or spermine). IL‐18 maintains intestinal barrier integrity through stimulation of production of anti‐microbial peptides (AMP) by Paneth cells (in dark pink), mucus synthesis by goblet cells (in green) and epithelial proliferation through induction of epithelial stem cells (in blue). In addition, IL-18 is also produced by intestinal neurons controlling homeostatic goblet cells AMP production. In this way, IL‐18 contribute to maintain a normal microbiota in the digestive tract. When commensal bacteria enter the mucosa, macrophages from the lamina propria secrete IL‐18, which participate to control infection. In pathological conditions (lower illustration), the intestinal epithelial barrier is disrupted, and microbes can enter in the lamina propria where they stimulate resident macrophages to produce IL‐18. This excess of IL-18 is deleterious to intestinal epithelial barrier integrity, leading to leukocytes recruitment from the blood and inhibition of mucus production by goblet cells destruction. Altogether, these injuries shift gut microbiota favoring dysbiosis. Some of the enriched bacterial populations lead to an increased influx of Toll-Like Receptor agonists into the portal circulation. Reaching the liver, theses noxious molecules lead to hepatic overexpression of TNF-α and others pro-inflammatory cytokines that drives NASH progression. Figure produced using illustrations from BioRender.com.

The physiological functions of epithelial/neuronal-derived IL-18 in the gut/microbiota interface, including its roles in the control of intestinal barrier integrity and the production of mucus and AMPs (all required to maintain a normal microbiota in the digestive tract) are illustrated in the upper panel of **Figure 2**. In contrast, the deleterious action of immune cells-derived IL-18 (consecutive to gut microbial invasion) leading to altered gut microbiota composition, increased TLR agonists in the portal circulation and NASH progression is shown in the lower panel of **Figure 2**.

### Effect of IL-18 on hepatic carcinogenesis

As a potent activator of NK cells, IL-18 could have a potential anti-cancer activity. This hypothesis has been validated by experimental observations in mice. Treatment with a combination of IL-18/IL-12 decreased tumor burden in mice with established HCC (158). In colorectal cancer with hepatic metastases, burden is exacerbated in NLRP3-deficient mice (159). Downstream NLRP3, IL-18 promoted the maturation of hepatic NK cells and triggered FasL mediated cytotoxicity (159, 160). Downregulated expression of NLRP3 inflammasome in HCC correlated with the aggravation of carcinoma, while reconstitution of NLRP3 inflammasome reversed the malignant phenotype of HCC (161). Nevertheless, in contrast to this protective role of NLRP3/IL-18 axis against hepatic tumor growth, other results showed that an inhibition of IL-18 signaling could protect TLR2^-/-^ mice from diethylnitrosamine (DEN)-induced carcinogenesis (162). The underlying mechanism seems to involve a limitation of IL-18-induced accumulation of myeloid-derived suppressor cells (162).

From a clinical point of view, several observations have corroborated this role for IL-18 in liver cancer progression. Expressions of IL-18 and IL-18R were upregulated in HCC tissue specimens (163, 164). IL-18 suppressed the apoptosis of human HCC cells (163) and promoted hepatoma cells metastasis and migration (164). The underlying mechanism could be partially attributable to the increased activities of ECM metalloproteinase (MMP)-2/3/9 by IL-18 (164). Circulating levels of IL-18 were elevated in patients with HCC compared to controls and they significantly correlated with the presence of venous invasion and advanced tumor stages (165). Finally, mutations in IL-18 alleles contributed to susceptibility to HCC and severity of the disease in general populations and in patients infected with hepatitis virus (166–171).

## Discussion

To conclude, research over the past 20 years has provided an increasingly complex view of the pleiotropic functions of IL-18. Beyond being a classic cytokine allowing fine-tuning of immune cells communication, this protein has emerged as a key regulator in the control of metabolism, in normal physiology, but also in pathological conditions such as obesity, diabetes and associated liver disorders.

Important metabolic functions resulting from physiological levels of IL-18 should clearly be dissociated from deleterious effects resulting from supra-physiological levels of IL-18 reached through immune stimulation (in a global pro-inflammatory context) or pharmacological administration (in an experimental context). Despite the significant advances in the understanding of physiological functions of IL-18 and its role in the occurrence and progression of metabolic diseases, several questions remain unanswered: What is the trigger that leads to IL-18 production by inflammasomes in metabolic diseases? Could different stimuli activate different inflammasomes, resulting in different production of IL-1 and IL-18? What is the respective contribution of different metabolic tissues (such as WAT and skeletal muscle) to circulating levels of IL-18? What are the molecular mechanisms underlying IL-18 resistance? Is there some link between leptin, insulin and IL-18 resistance? How circulating levels of IL-18 are related to IL-18 tissular actions? Can different cytokines (as IL-37 in humans) modulate IL-18/IL-18R interaction, and if so, how?

Answering these important questions will reinforce our understanding of IL-18 metabolic roles. In addition, more pre-clinical studies involving IL-18 regulatory steps, such as maturation by inflammasome, retention by IL-18BP or antagonism by anti-IL-18 or anti-IL-18R antibodies could open new therapeutic options for patients with metabolic diseases such as obesity, diabetes, and NAFLD/NASH.

## Author contributions

ES contributed to the literature search and original draft preparation. ES and FJ contributed to text review and editing. All authors have read and agreed to the published version of the manuscript.

## Funding

This work was funded by the Swiss National Science Foundation (SNSF grants 189003), the Swisslife Foundation, the Fondation pour l’innovation sur le cancer et la biologie and the Vontobel Foundation. Open access funding was provided by the University of Geneva.

## Conflict of interest

The authors declare that the research was conducted in the absence of any commercial or financial relationships that could be construed as a potential conflict of interest.

## Publisher’s note

All claims expressed in this article are solely those of the authors and do not necessarily represent those of their affiliated organizations, or those of the publisher, the editors and the reviewers. Any product that may be evaluated in this article, or claim that may be made by its manufacturer, is not guaranteed or endorsed by the publisher.
