# Erratum: Interleukin-18 in metabolism: From mice physiology to human diseases

**DOI:** 10.3389/fendo.2023.1175361

**Published:** 2023-03-09

**Authors:** Frontiers Production Office

**Affiliations:** Frontiers Media SA, Lausanne, Switzerland

**Keywords:** obesity, diabetes mellitus, NAFLD, NASH, gut microbiota, inflammation, interleukin-18

An omission to the funding section of the original article was made in error. The following sentence has been added: “Open access funding was provided by the University of Geneva”.

The original version of this article has been updated.

